# Response to “Hypercapnic respiratory failure with insufficient response to fixedlevel PS-NIV: Is AVAPS the end solution?”

**DOI:** 10.5578/tt.20239924

**Published:** 2023-06-13

**Authors:** N. A. A. ÖZTÜRK, Ö. A. GÜÇLÜ, E. DEMİRDÖĞEN, A. G. DİLEKTAŞLI, S. MAHARRAMOV, F. COŞKUN, E. UZASLAN, A. URSAVAŞ, M. KARADAĞ

**Affiliations:** 1 Department of Pulmonology, Uludağ University Faculty of Medicine, Bursa, Türkiye

Dear Editor,

We appreciate the interest in our article describing the successful use of AVAPS mode in patients with insufficient response to PS-NIV and your comments. We would like to address the questions and concerns about our study.

Authors pointed an important issue concerning B-type natriuretic peptide (BNP) measurements. BNP is indeed a valuable marker for diagnosis of heart failure and hypervolemia. ESC Heart Failure Guideline recommends using BNP measurements as a step for diagnosis of heart failure but states that current evidence does not support routine measurements in follow-up to guide the treatment ( 1 ). We didn’t evaluate repeated BNP measurements as it is not recommended. Vallabbhajosyula et al. evaluated the role of BNP measured at admission among patients with acute exacerbation of COPD with or without hypercapnic respiratory failure. Serum levels above 500 pg/mL of BNP compared to lower levels of BNP, is related with non-invasive ventilation (NIV) failure and invasive mechanical ventilation need ( 2 ). Within our study population, patients needing AVAPS mode NIV treatment presented similar frequency of left heart failure and similar BNP measurements at the time of admission, as stated in Table 3 in the main article. Therefore we concluded serum BNP levels in our population does not create a major effect in terms of PS-NIV response.

Basic characteristics of the study population such as severity of COPD and obesity, body position under treatment is also mentioned in the “letter to editor”. All patients were in a semi-recumbent position (15°-45°) while using NIV devices enabling comparison between body positions. Patients having respiratory insufficiency due to obesity hypoventilation were excluded from the study. After excluding morbid obese patients unfortunately we didn’t evaluate the effects of body weight seperately. On the other hand, previous study conducted by Türk et al. concluded obesity and body positioning didn’t influence the course of PaCO2 under NIV treatment ( 3 ). Our analyses revealed that in terms of pulmonary function test there is no statistical difference between two groups. FEV1 %pred in patients with insufficient response was 25.5 (7.5-36.0) compared to 32.5 (23.3-57.5) in patients using only PS-NIV mode (p= 0.34). However, missing values from the study population and different intervals between the exacerbation and functional evaluation creates a difficulty for unbiased interpretation. Therefore, the results are not added to the original manuscript.

The authors raise an important question about the improvement of sleep quality. Our primary goal was to evaluate the benefit AVAPS mode on reduction in PaCO2. Therefore, we didn’t evaluate the sleep efficiency and related quality of life. However, studies comparing effect of AVAPS mode in obesity hypoventilation syndrome evaluating sleep efficacy have conflicting results. While in some studies patients preferred AVAPS mode ( 4 ) patients who were accustomed to PL-NIV also reported worse quality of sleep under volume targeting NIV ( 5 ).

As suggested by the authors and presented in our limitations; it might be beneficial to evaluate parameters such as data of leaks, breathing frequency and delivered tidal volume from NIV device, pulmonary function measurements, diaphragm functions within a larger sample to further conclude the effects of AVAPS mode compared to PS-NIV in patients with insufficient response.
